# A Case of Lithotomy in the Female—Double Calculus

**Published:** 1845-06

**Authors:** B. W. Groce

**Affiliations:** Talladega County, Alabama


*A Case of Lithotomy in the Female—double Calculus.* By B. W. Groce, M. D., of Talladega County, Alabama.—During the month of April, 1843, I was called to visit Mrs. N. S., (aged 23 years, and of leuco-phlegmatic temperament,) in consultation with Dr. Sumners, for the purpose of removing stone in the bladder, by an operation. I found Mrs. S. in an extremely debilitated condition. She informed me that she had been suffering from the effects of the stone for several years. She had taken various medicines without producing any more than mere temporary relief; and was at this time labouring under the distressing effects of dyspepsia. On further inquiry, she informed me that in childhood she had been very much troubled with asthma, but which pretty much subsided about the age of puberty. She did not, however, long enjoy the consolation of having gotten rid of this disease, before one of an equally distressing character made its appearance, to wit, amenorrhœa; under which she laboured until near the age of nineteen. Upon the appearance of her catamenia she began to experience symptoms of gravel, which continued to increase in violence until she was happily relieved by an operation. On my arrival, Dr. S. represented the stone as being about three-fourths of an inch from the external orifice of the urethra. Upon making a minute examination, I discovered this to be the case, and that the stone was so large as to prevent the passing of the finger up the vagina. Deeming dilatation impracticable, we immediately determined to operate; which was performed by making an incision (with the smallest scalpel in the dissecting case,) through the vagina and urethra, immediately upon the stone. After completing the incision, the dressing forceps (in the pocket case) were introduced, and the stone grasped; but discovering that it could not be extracted without considerable effort, the finger was inserted, and the calculus raised from its bed, near the internal orifice of the urethra, (for it had been so long in this situation that it had become pretty firmly attached to the mucous membrane,) and then easily removed.

In a few moments after the operation the patient was attacked with rigors, but which subsided immediately upon the administration of a little camphor water. She soon fell into a quiet and pleasant sleep, and rested well during the after part of the day and that night. I visited her the second day after the operation and found her doing well: pulse 85, in no pain, the incision was healing by the first intention, and the urine passing off by the natural channel.

I heard nothing more of our patient (as I lived at some distance,) until about eight days after, when I was unexpectedly summoned to see her again. When I arrived, I found Dr. Sumners already in attendance, who stated that another calculus, fully as large as the first, had come down, and occupied pretty much the same position as the one already removed. I was indeed astonished; but it immediately occurred to me that we had neglected the important and necessary precaution of sounding the bladder after the operation.

We determined to make a second effort to relieve the sufferings of our patient, whose pains had now become almost intolerable. Upon making a vaginal examination, we found the previously made incision partly reopened; this we enlarged, then introduced the forceps, and removed the stone without difficulty. The bladder was now thoroughly explored, and no other stone being detected, she was placed quietly in bed, and the strictest orders given as to her regimen, &c.

She recovered from this second operation without a single unfavorable symptom, except incontinence of urine, which, for some time, threatened to be very obstinate. This, however, was finally relieved by astringent injections, bathing, &c.; and I am now happy to say, that she has since been delivered of a fine, healthy boy, and is at this time enjoying unusually good health.

The calculi are of the mulberry character; each half as large as a pullet's egg, and weighing something over an ounce. The one first removed is of an oval shape, with the upper surface smooth and polished; produced, I suppose, from the urine passing over it;* the other part of the stone is exceedingly rough. The second stone is round, and rough over its entire surface.—*Ibid.*

* Most probably from friction of the second calculus, which latter became rough over its entire surface in the eight days after the removal of the first.—Eds.

